# Relevance of the Hoover´s signal and the Babinski´s test for the diagnosis of functional hemiparesis

**DOI:** 10.22088/cjim.15.2.364

**Published:** 2024

**Authors:** Otto J Hernandez Fustes, Carlos Arteaga Rodriguez

**Affiliations:** 1 Complexo Hospital de Clínicas da Universidade Federal do Paraná, Serviço de Neurologia, Hospital de Clínicas, Curitiba PR, Brasil; 2 Universidade Positivo, Curitiba, PR, Brasil

**Keywords:** Functional hemiparesis, Hoover`s sign, Babinski test

**Dear Editor**

Patients presenting complex functional disorders become more frequent, especially in the last decades. They are patients who demand great amounts of resources with expensive and unnecessary exams, and many times they accuse the health professionals for not deciding them complaints. 

The first case of described factitious disorder its from 1783 to 1785 in the General Nottingham Hospital of England, where a woman named Kate Hudson mysteriously presented needles, nails and pieces of bones under her skin. According to Eisendrath, there are three situations that are characterized by factitious disorders. They are: simulation, where the patient is aware that produces the symptoms and their reasons; Syndrome of Münchausen, where it has conscience of the production of the symptoms for the patient, however this is unconscious of the reasons; and hysteria, where there is as unconsciousness of the production as the motivation of the picture. (1) 

The definition of symptoms and/or self-imposed factitious signs were considered according to the diagnostic criteria of DSM 5 as follows: A. Falsification of physical or psychological signs or symptoms, or induction of injury or associated disease; the identified fraud. B. The individual presents himself / herself to others as sick, disabled or injured. C. Fraudulent behavior is evident even in the absence of obvious external rewards. D. The behavior is no longer best explained by another mental disorder, such as delusional disorder or another psychotic condition. (2)

The objective is to show the importance of clinical history and neurological examination Hoover´s signal and the Babinski´s test for the diagnosis of functional hemiparesis. The study was waived by the Research Ethics Committee due to its observational, non-interventionist and descriptive nature, without nominal identification. We observed the ethical principles contained in the Resolution of the National Health Council n. 466 of December 12, 2012. Two men and a woman, with 30, 32 and 45 years old, who were admitted to the neurology service for acute hemiparesis with initial clinical suspicion of cerebrovascular disease who were carried out, in addition to laboratory tests, cardiological tests, Doppler and carotid echography, neuroimaging tests (CT and MRI) that showed no brain lesions and that on the neurological examination they presented Hoover's sign and positive Babinski's test, with no other changes in the exam, confirming functional hemiparesis. 

Functional neurological manifestations are varied and, in the literature, patients with apraxia, seizures, meningism, even long-term paraplegia as a way of presentation. (3, 4) The related motor deficit to this disorder is very rare; however, its diagnosis can be made clinically, as much for anamnesis, neurological examination with specific maneuvers and evolution of the clinical picture. It was demonstrated in our patients. 

Functional weakness is recognized by non-severe variability over time and discordant performance between assessments, especially during the same examination, it may be global or limited to one side of the body, mimicking a stroke. (5) Somatoform unilateral weakness simulating true vascular stroke is a rare presentation in adults – a total of 31 patients have been reported. (6)

Important signals were described for Babinski, that is considered one of the most important neurologists to describe signals that differentiate a factitious disorder of organic one, related the motor deficit. (7) When the patient is requested to move affected members, the movements tend to be slow, hesitates, frequently with contraction of the agonistic and antagonistic muscles of simultaneous and intermittent form. This sample that the movements are affected, instead of individual muscles. When it is asked to the patient to press the fingers of the examiner, there is "disuse of the effort" when the muscles of the face, shoulder and arm contract, but the fingers stand still and limp. (7) 

Hoover described his sign in 1908, is based on associated movements in the opposite leg. When a person flexes a hip, the contralateral hip is extended. It is assumed that this is a result of the crossed extensor reflex, examiner takes the heel of the lying patient, and asks to him to raise the leg not paralyzed. The examiner will feel a pressure for low against the hand in the supposed paralyzed leg. The sign relies on the principle of synergistic contraction. Involuntary extension of the “paralyzed” leg occurs when flexing the contralateral leg against resistance. It has been neglected, although it is a useful clinical test. The patient lies supine, the examiner’s hand is placed under the non-paralyzed heel, and the patient is asked to elevate the paralyzed leg. In organic paresis the examiner feels a downward pressure under the non-paralyzed heel; in malingering no pressure is felt. However, some have used it in a less precise context as a sign of pain or weakness in the back or lower extremities. (8) Hoover`s sign is the most useful test for functional weakness and the only one that has been subjected to scientific study with a neurological control group. (9)

There is also the Babinski trunk-thigh test, described in 1897, where the lying patient is requested to sit down with his arms crossed ahead of the thorax. Normally the heels are pressured against the bed. In organic hemiplegia, the paretic member rises involuntary because he cannot force the heel for low. In the hysterical hemiplegia, the healthy leg can be raised while "paralyzed" is pressured against the bed. In the described cases, the absence of signals of first neuron disease, as the amplified reflexes, Babinski signal in the same side, the evolution of complete or partial improvement in a short interval of time without physical therapy aid, beyond negative examinations of image excludes organic etiology. (8) 

The factitious disorder is a psychiatric condition, diagnosed when there is intentional production or simulation of physical or psychological signs or symptoms where the incentive is to assume the sick role and external incentives for behavior are absent. In the ICD-10, it is defined as a repeated and consistent feigning of symptoms with an obscure motivation for the behavior and best interpreted as a disorder of the behavior of the illness and the patient's role. (10). When excluding organic causes, the main differential diagnoses are simulation and somatoform disorders, in which both unconscious production of symptoms and unconscious motivations are present. In conclusion, although uncommon, the motor deficit as symptom of a functional pathology, it is necessary to be intent for the diagnosis. This is important to break the vicious circle of internments and examinations, many times invasives, providing to the patient appropriate therapeutical and minimizing unnecessary expenses of the institutions. Thanks to the emphasis given to this subject previously, today we can count on maneuvers for this end, whose use becomes easy to differentiate between an organic and functional etiology. 
